# Supplementary material for: Drivers of inappropriate use of antimicrobials in South Asia: A systematic review of qualitative literature
Source: PLOS Glob Public Health. 2024 Apr 4;4(4):e0002507. doi: 10.1371/journal.pgph.0002507 (PMC10994369; doi:10.1371/journal.pgph.0002507)
Supplement: Attachment — Submitted filename: Rebuttal letter_final.pdf [file pgph.0002507.s003.pdf]

Response to reviews
Manuscript No. PGPH-D-21-01910

Dear Editor and Reviewers,

We would like to thank you for your time to review our manuscript ***Drivers of inappropriate use of antimicrobials in South Asia: A systematic review of qualitative literature***. We appreciate your thoughtful review and the opportunity given to revise and resubmit this paper. We have thoroughly considered each of the reviewer comments, which we believe substantially improve both the quality and clarity of the manuscript. Please find below our responses to the comments. Both the track-change copy of the manuscript and the clean version of the manuscript have been uploaded.

We look forward to your review of this revised manuscript and to the possibility of having it published in *PLOS Global Public Healt*h.

Reviewer #1:

1. **I want to thank the authors for writing a review on this hidden pandemic (AMR) in South Asia.**

   We thank the reviewer for this positive feedback.

2. **Abstract: Conclusion is missing. Also, consider using a structured format for abstract writing in this scenario.**

   Per journal guidelines, we will keep the abstract conceptually divided into three sections (Background, Methodology/Principal Findings, and Conclusions/Significance), but will not apply these distinct headings to the Abstract within the file.

   We have updated the abstract to include a more defined concluding statement, both summarizing the findings as well as their implications:

   > *"This study is the first to succinctly identify a range of norms, behaviors, and policy contexts driving inappropriate use of antimicrobials in South Asia, emphasizing the importance of working across multiple sectors to design and implement approaches specific to the region."*

3. **Introduction: Well written.**

   Thank you for this positive feedback.

4. **Methods: Instead of overview, I suggest authors say overview and study criteria.**

   We agreed with this feedback and have changed the subtitle to "Overview and study criteria."

5. **Methods: You mention only articles published in English were included, and is that not a bias?**

This is certainly one of the limitations of the study, which are detailed in the last paragraph of the discussion. We have expanded on this statement and described the potential impact by including the following:

> *"We only included studies published in English, potentially excluding studies that are otherwise eligible; however, we did not identify any manuscripts in the two databases that were published in a language other than English."*

6. **Methods: What was the review period? Who did the data search, when, how were full articles accessed if not open access, and why were only two databases searched?**

   The initial search was conducted on September 18, 2023, which is stated under "Search strategy and selection criteria." All searches and reviews were initially done by the first author (JLM), and repeated by the third author (ORH) for consistency.

   We have updated the manuscript to include this information under "Search strategy and selection criteria":

   > *"On September 18, 2023, we conducted a systematic search of the electronic databases PubMed and Embase. The search and selection processes were conducted by the first author (JLM) and repeated by the third author (OH) for consistency."*

   We chose Embase and PubMed to maximize search completeness while keeping our methodology simple and replicable. PubMed, provided by the US National Library of Medicine, includes MEDLINE with records indexed by MeSH terms (which were utilized during our search), as well as older records prior to the inception of MEDLINE and records from journals not included in MEDLINE. PubMed alone yields a very comprehensive search in itself, but we opted to also search Embase due to its even larger size and access to European and Asian journals not included in MEDLINE. With these two databases used together, we are confident we yielded a complete search.

7. **Methods: How were mixed-method studies handled?**

   All eligible mixed-method studies had qualitative and quantitative results separated in their findings, allowing us to extract only the qualitative data from eligible South Asian countries. We have updated the manuscript to state the following under "Data extraction and synthesis":

   > *"Eligible mixed-methods and multi-site studies were adjusted to only include qualitative findings from World Bank defined South Asian countries in our analysis."*

8. **Methods: Critical appraisal should come after data extraction and how was consensus done for discrepancies?**

   We agree with this feedback and have re-ordered the sections. Consensus was reached for discrepancies upon consulting both the first (JLM) and last (MHW) authors. We have updated the manuscript to state the following under "Critical appraisal":

"To assess the quality of the included studies, two individuals evaluated each paper using the Critical Appraisal Skills Program (CASP) for qualitative studies and resolved any discrepancies by consensus upon consulting the first (JLM) and last (MHW) authors."

9. **Methods: The quality check stage is missing from the PRISMA table [figure].**

   We have updated the PRISMA flow chart (Figure 1) to include this step in our search process.

10. **Methods: Table 3 - I recommend a column added stating main findings of the studies included in the final review.**

    We agree with this feedback and have updated the table to include a summary of themes that emerged in each of the included study. These changes may be viewed in the track-change copy of the revised manuscript.

11. **Results: Included studies - Can authors explain what happened at each stage of the PRISMA table and why?**

    This comment is not clear. The supplemental table with the PRISMA checklist (Supplement 1) indicates where in the manuscript to find the required elements of a systematic review. If the reviewer requires additional information, we can respond.

<u>Reviewer #2:</u>

1. **Thank you for the opportunity to review this manuscript. I want to commend the authors for taking on this important topic. However, I have some comments and questions.**

   We thank the reviewer for their positive feedback and for taking the time to review our manuscript.

2. **Methods: In my opinion, the topic can benefit from a scoping or narrative review instead of systematic review.**

   At the conception of this paper, we discussed the possibility of conducting a scoping review rather than a systematic review. As the reviewer mentions in later comments, the findings of these individual studies (i.e., the factors driving antimicrobial stewardship) are becoming well defined in their respective countries. This allowed us to form a specific research question, search criteria, and geographic area of interest in which we can synthesize and report these findings. Prior systematic reviews have synthesized qualitative antimicrobial studies in other regions of the world, such as sub-Saharan Africa (i.e., Porter et al, *A systematic review of qualitative literature on antimicrobial stewardship in Sub-Saharan Africa*. Glob Health Res Policy. 2021). We determined that it would be appropriate to do a systematic review of qualitative data in South Asia, where antimicrobial resistance is rising.

3. **Methods: Inclusion of grey literature such as government reports can be beneficial as they may also contain some valuable information.**

   We discussed this possibility when forming our search and selection methodologies and considering databases. With a rising interest in antimicrobial resistance and stewardship research as well as qualitative methodologies, there have been several studies published in recent years. We were confident that limiting the search to peer-reviewed, original research publications would provide us with plenty of data to include in our review and reach thematic saturation. In the future, it might be valuable to conduct a separate review specifically focused on South Asian policy, legislature, and government reports.

4. **Methods: The rationale regarding what this research adds to the knowledge is not clear. Other than the setting, the findings may not be new and already known. Other studies in LMICs have found similar results.**

   Over the past decade, qualitative research investigating antimicrobial resistance and stewardship has increased in number, offering important insight into addressing resistance. This study is the first to synthesize and report qualitative findings specific to South Asia, which has been done in other regions of the world such as sub-Saharan Africa (i.e., Porter et al, *A systematic review of qualitative literature on antimicrobial stewardship in Sub-Saharan Africa*. Glob Health Res Policy. 2021). Additionally, this study is the first to report findings utilizing the social ecological framework. Antimicrobial resistance is a public health crisis with opportunity for prevention and intervention at the patient, provider, facility, community, and government levels. Therefore, the use of this framework is not only innovative but critical to facilitating stewardship.

5. **Methods: For search terms- why 'drug resistance' was used instead of 'antimicrobial resistance', which is used more commonly in this field? more synonyms of 'antimicrobial stewardship' to broaden search terms may increase the original pool of articles.**

   We chose our search terms based on both databases' indexed terminology, which are designed to also capture synonyms and related terms. For PubMed, we chose our search terms based on MEDLINE MeSH terms as classified by the National Institutes of Health and US National Library of Medicine. These terms were also searched as plain text to ensure we capture studies that were not properly indexed. For Embase, we similarly chose Emtree terms designated by Elsevier and searched these terms as both Emtree indexed and free text keywords. Together, we are confident these terms adequately capture all eligible studies.

6. **Methods: Inclusion of CINAHL, MEDLINE etc. libraries may offer more comprehensive literature search, which may have more qualitative articles than PubMed and Embase.**

   We chose Embase and PubMed to maximize search completeness while keeping our methodology simple and replicable. PubMed, provided by the US National Library of Medicine, includes MEDLINE with records indexed by MeSH terms (which were utilized during our search), as well as older records prior to the inception of MEDLINE and

records from journals not included in MEDLINE. PubMed alone yields a very comprehensive search, but we opted to also search Embase due to its even larger size and access to European and Asian journals not included in MEDLINE. While there are many other great databases to search, we are confident we yielded a complete and replicable search.

7. **Methods: How findings were extracted when articles included multiple sites (e.g., studies across LMICs, studies including South Asia and another region)?**

   All eligible multi-site reported their findings by specifying the country in which they applied to, allowing us to extract only the data from World Bank designated South Asian countries. We have updated the manuscript to state the following under "Data extraction and synthesis":

   "Eligible mixed-methods and multi-site studies were adjusted to only include qualitative findings from World Bank defined South Asian countries in our analysis."

8. **Results: Finally, the results section can be broken down in subtopics to help the reader in understanding sub themes within broader themes.**

   We agree with this feedback and have separated the results section into subcategories. These changes may be viewed in the track-change copy of the revised manuscript.
