# Supplementary material for: Drivers of inappropriate use of antimicrobials in South Asia: A systematic review of qualitative literature
Source: PLOS Glob Public Health. 2024 Apr 4;4(4):e0002507. doi: 10.1371/journal.pgph.0002507 (PMC10994369; doi:10.1371/journal.pgph.0002507)
Supplement: Attachment — Submitted filename: Response to Reviewers Rebuttal Letter_092723.docx [file pgph.0002507.s002.docx]

**Response to review of Manuscript #PGPH-D-23-01577**

This is a resubmission of Manuscript #PGPH-D-23-01577, which was submitted on August 13, 2023. Please see below the editor’s comment and our response.

**Review: We are concerned that the literature review was completed in July 2022. Systematic and scoping reviews should provide an up-to-date overview of the research topic covered. Since the literature search was completed over a year ago, we are concerned that this study does not meet this requirement. As a result of these concerns, we cannot consider the manuscript for publication at PLOS Global Public Health at this time. However, if you are able to address the concerns discussed above, we may be able to consider a revised version of the manuscript.**

Response: We reran the search on September 8, 2023. The search resulted in 25 new papers that were identified, with six meeting the study criteria. These six papers were incorporated into our results and discussion, augmenting our initial findings and providing additional insight. We appreciate the opportunity to resubmit this paper for consideration.
